# Early-Life Human Microbiota Associated With Childhood Allergy Promotes the T Helper 17 Axis in Mice

**DOI:** 10.3389/fimmu.2017.01699

**Published:** 2017-12-01

**Authors:** Dagbjort H. Petursdottir, Sofia Nordlander, Khaleda Rahman Qazi, Claudia Carvalho-Queiroz, Omneya Ahmed Osman, Eva Hell, Sophia Björkander, Yeneneh Haileselassie, Marit Navis, Efthymia Kokkinou, Ivan Zong Long Lio, Julia Hennemann, Björn Brodin, Douglas L. Huseby, Caroline Nilsson, Diarmaid Hughes, Klas I. Udekwu, Eva Sverremark-Ekström

**Affiliations:** ^1^Department of Molecular Biosciences, The Wenner-Gren Institute, Arrhenius Laboratories for Natural Sciences, Stockholm University, Stockholm, Sweden; ^2^Department of Medical Biochemistry and Microbiology, Uppsala University, Uppsala, Sweden; ^3^Department of Clinical Science and Education, Södersjukhuset, Karolinska Institutet and Sachs’ Children’s Hospital, Stockholm, Sweden

**Keywords:** infant microbiota, allergic heredity, immune development, germ-free, T helper 17-responses

## Abstract

The intestinal microbiota influences immune maturation during childhood, and is implicated in early-life allergy development. However, to directly study intestinal microbes and gut immune responses in infants is difficult. To investigate how different types of early-life gut microbiota affect immune development, we collected fecal samples from children with different allergic heredity (AH) and inoculated germ-free mice. Immune responses and microbiota composition were evaluated in the offspring of these mice. Microbial composition in the small intestine, the cecum and the colon were determined by 16S rRNA sequencing. The intestinal microbiota differed markedly between the groups of mice, but only exposure to microbiota associated with AH and known future allergy in children resulted in a T helper 17 (Th17)-signature, both systemically and in the gut mucosa in the mouse offspring. These Th17 responses could be signs of a particular microbiota and a shift in immune development, ultimately resulting in an increased risk of allergy.

## Introduction

The development of allergy is dependent on changes in how the immune system encounters and interacts with the environment in a genetically predisposed individual. The microbiota is considered to play an important part in these processes and it can be observed already *in utero*. A recent murine study revealed that even transient bacterial colonization of the pregnant female affects immune properties in the offspring, such as enhancing intestinal barrier properties (1). At birth and in early childhood drastic immunological changes occur at mucosal surfaces due to extensive microbial colonization. At this developmental stage, it is imperative that tolerance is induced and dysregulated immune responses are considered to contribute to the development of allergy (2). Increased differentiation of T helper 2 (Th2) cells, a tissue environment rich in the cytokines IL-4, IL-5, IL-9, and IL-13, and suppressed Th1 cell development, are traits classically associated with allergy (3). The cellular interactions causing sensitization and allergy have been investigated in animal models. In the case of food allergies, altered functions of dendritic cells and intestinal epithelial cells (IEC), as well as gut barrier properties, have been suggested to affect disease outcome (4).

The infant intestinal microbial communities are known to fluctuate during the first years of life. A number of studies focusing on children with allergic heredity (AH) reported correlations between reduced early-life intestinal microbial diversity and elevated risks of allergic sensitization and allergic rhinitis (5), and eczema (6–9). Efforts to manipulate the microbiota in order to generate a more favorable composition have so far not yielded clear results. Multiple studies have examined the administration of probiotic bacterial strains to infants with AH in order to reduce the development of allergy. Some beneficial effects have been reported with regards to reduced incidence of eczema after administration of *Lactobacillus (L.) reuteri* (10) or a combination of a prebiotic and several probiotic strains (11). Still, probiotics do not induce major shifts in the intestinal microbiota. A study describing the infant fecal microbiome during supplementation with different probiotic species showed that most changes in bacterial composition could be correlated to increased age (12). This highlights the complexity of simultaneously considering both the microbiota that might act allergy-protective, and the developmental stage which is crucial for immune system skewing.

We have previously described the beneficial effects on allergy development of early-life colonization with group I lactobacilli (*L. casei, L. paracasei*, and *L. rhamnosus*) in two separate prospective birth cohorts (13, 14). Importantly, early-life colonization with the group I lactobacilli was correlated with a reduced risk of actually developing allergic diseases by the age of 5 years also in children with AH (14). This would suggest that certain types of microbiota could provide an environment less favorable to IgE sensitization and subsequent allergy. In this study, we used what previously has been termed a human microbiota-associated mouse model (15) to investigate how different types of human infant microbiota would affect immune system development. Cellular composition of the immune system and T- and B-cell responses were evaluated in the progeny of germ-free (GF) mice inoculated with human microbiota. We identified that microbiota associated with allergy later in childhood induced increased CD4^+^ T-cell RORγt expression, and elevated production of IL-17A and IgA, both at local intestinal and systemic sites, indicating expanded Th17 responses.

## Materials and Methods

### Ethics Statement

The part of the study involving human material was carried out in accordance with the recommendations of the Human Ethics Committee at Huddinge University Hospital, Stockholm, and the parents provided their informed verbal consent. No written documentation of the participants’ informed approval was required, which was agreed to by the Human Ethics Committee and was according to the regulations at the time of the initiation of the study.

The murine study was approved by and carried out in accordance with the recommendations of the Regional Animal Research Ethical Board, Stockholm, Sweden.

### Children Cohort

The fecal samples were collected from children participating in a prospective birth cohort of children with different AH (16). From a subgroup of these children, fecal samples were analyzed for the presence of lactobacilli in feces during early life (14). The samples were collected between 2 and 8 weeks after birth and the bacterial content screened. Techniques for collection and microbiota analysis of the human infant fecal samples have been reported previously (13, 14). The children were subdivided based on history of atopic disease in the immediate family [AH or no allergic heredity (NH)], and on the presence of lactobacilli type I in feces at the time of, or close to sampling (L). For the human microbiota-associated mouse model, individual infant fecal samples were randomly selected from three different groups of children, previously associated with a low- or high-allergy risk (14): the no allergic heredity, lactobacilli+ (NH_L) group (low risk) (*n* = 7), allergic heredity, lactobacilli+ (AH_L) group (low risk) (*n* = 6), and AH group (high risk) (*n* = 6), pooled within each group and used as the initial inoculum. In the NH_L and AH_L groups, a minority (1/7 and 1/6, respectively) of children became allergic. All (6/6) children providing samples for the AH pooled material developed allergy.

### Mice

Work with axenic and conventionalized C57BL/6 mice was carried out at the Core Facility for Germfree Research at the Karolinska Institutet, Stockholm, Sweden. Mice were kept in sterile isolators and were fed autoclaved feed (R36, Lantmannen, Sweden) and water *ad libitum*. Mice were colonized with human infant microbiota at 6 weeks of age. Infant fecal samples were diluted in sterile phosphate-buffered saline (PBS) (0.3 g/ml) and brought to a homogenous solution. The mice were then orally gavaged with 0.1 ml of fecal PBS solution. After 6 weeks, mice were bred. A booster gavage was administered to the females on gestational days 15–17. The offspring were sacrificed at 7–8 weeks of age. The fat to lean mass body composition was measured by magnetic resonance imaging (MRI) technique (EchoMRI-700/100 Whole Body Composition Analyzer, Echo Medical Systems).

### Cell Isolation

Spleens and Peyer’s patches (PP) were removed and passed through a cell strainer (90 µm, Falcon) to obtain a single cell suspension. Splenocytes were treated with red blood cell lysing buffer (Sigma-Aldrich), washed and resuspended in complete medium: RPMI 1640 with FCS (10%, Gibco) and penicillin–streptomycin (100 U/ml and 100 µg/ml, respectively, HyClone). Cells from PP were cultured in complete medium but with additional gentamicin (50 µg/ml, Gibco) to prevent bacterial contamination. After isolation, cells from spleen and PP (3–5 × 10^6^ cells/ml) were either left unstimulated or polyclonally stimulated with αCD3 (1 µg/ml, Biolegend) and αCD28 (5 µg/ml, BioLegend) for 24 h. Cell culture supernatants were harvested and stored at −80°C.

Colonic lamina propria (cLP) leukocytes were isolated as previously described (17). In brief, colon tissue was incubated with EDTA (5 mM) to eliminate epithelial cells, followed by digestion with collagenase 8 (0.5 mg/ml, Sigma Aldrich) and DNase 1 (40 µg/ml, Roche). The released cells were layered on a 30/40/70% Percoll gradient (GE Healthcare).

### Flow Cytometry

Cells from PP, colon, and spleen were treated with Ultra-LEAF anti-mouse CD16/32 (Clone 93, BioLegend) and subsequently stained with antibodies against surface markers: CD45-A488 (30-F11), CD3ε-BV421 (145-2C11), CD4-APC/Cy7 (GK1.5), CD25-PE/Cy7 (PC61), and CD304 (Neuropilin-1)-PE (3E12) (all from BioLegend). Intracellular staining for transcription factors was performed by fixing and permeabilizing cells using True nuclear buffer kit (BioLegend) and subsequent blocking with normal mouse serum and staining with anti-FOXP3-APC (150D, BioLegend) and RORγt-PerCP-Cy5.5 (Q31-378, BD Biosciences). Intracellular staining of cLP leukocytes was performed using the Foxp3 Staining Buffer Set (eBioscience). T-cell populations were characterized based on forward and side scatter properties and CD45 expression. For B-cell analysis, single cells isolated from PP and spleen were stained using the following antibodies: CD45-A488 (30-F11), B220-APC/Cy7 (RA3-6B2), CD21/CD35-APC (7E9), IgM- PerCP/Cy5.5 (RMM1), IgA-biotin (RMA-1), and BV421-streptavidin (all from BioLegend).

To distinguish live cells LIVE/DEAD Fixable Aqua Dead Cell Stain Kit (Invitrogen) was used. After staining, cells were acquired on an FACSVerse flow cytometer and data analyzed using FlowJo software.

### Measurement of Cytokines, IgG, and IgA

Cytokines were measured in cell culture supernatants using a mouse Luminex panel (Affymetrix eBioscience) according to the manufacturer’s instructions. The levels of IgG in serum and IgA in serum and small intestinal content were measured by enzyme-linked immunosorbent assay (ELISA) with commercially available ELISA kits (Mabtech).

### Quantitative Real-time PCR Analysis

Sections of distal small intestine and mid-colon were snap-frozen at necropsy. Tissues were disrupted using a homogenizer (MP Biomedicals) and total RNA was isolated using the RNEasy mini kit (Qiagen). cDNA synthesis was performed with the Superscript III system (Invitrogen) and messenger RNA (mRNA) expression of housekeeping gene *Hprt* (F: 5′-CCCAGCGTCGTGATTAGC-3′, R: 5′-GGAATAAACACTTTTTCCAAATCC-3′), *Reg3g* and *Retnlb* (QuantiTect Primer assays, Qiagen) was measured by SYBRgreen qPCR using KAPA SYBR FAST Universal qPCR kit (KAPA Biosystems) and a LightCycler 480 (Roche Life Science). Results were calculated using the 2^−Δ*C*(*t*)^ method.

### Immunofluorescence Staining

Distal small intestine was embedded in O.C.T. compound (VWR chemicals) and cut in 10 µm sections. Sections were stained with 1° antibodies mouse anti-E-cadherin (1.25 µg/ml, clone 36/E-Cadherin, BD Biosciences) and rabbit anti-Ki67 (1/200, clone SP6, Abcam) and 2° antibodies goat anti-mouse Alexa Fluor 488 and goat anti-rabbit Alexa Fluor 594 (4 µg/ml, both from Abcam) and DAPI (1/2,000, Cat No. D1306 Invitrogen). Sections were mounted with ProLong Gold antifade reagent (Molecular Probes). Images were acquired on an LSM 780 confocal microscope (Zeiss) using the 20× magnification objective.

### Statistics

The non-parametric Kruskal–Wallis test with Dunn’s multiple comparisons test was performed. GraphPad Prism software, version 7, was used. Data are displayed with median values or alternatively with mean ± SD. Results were considered to be statistically significant if *p* < 0.05.

### Sequencing and Amplification

#### 16S rRNA Sequencing

DNA was extracted using ZP Faecal DNA MiniPrep Kit (Zymo Research) and then purified with AMPure XP Beads (Beckman Coulter) according to the manufacturer’s guide. The 16S rRNA gene was amplified using barcoded 341 and 805 F primers (18) targeting the V3 and V4 regions of the gene. PCRs were performed in 50 µl volume using 1 U Phusion high-fidelity DNA polymerase (Thermo Fisher Scientific), 0.20 µM primers, 200 µM dNTP mix, and 2.5 mM MgCl_2_. The thermal program was the following: denaturation at 98°C for 30 s, 30 cycles of 98°C for 10 s, 55°C for 15 s, 72°C for 30 s, and final elongation at 72°C for 5 min. To verify the amplicon size, 5 µl of the PCR mixes were loaded on a 1.5% agarose gel stained with GelRed (Biotium). Amplicons were then purified with AMPure XP Beads (Beckman Coulter) according to the manufacturer’s protocol. Concentrations were estimated using Qubit Broad Range ds DNA reagents (Invitrogen). Barcoded samples were pooled in equimolar amounts, followed by adapter ligation using TruSeq DNA PCR-free LT Library Preparation Kit (Illumina). Agilent 2100 BioAnalyser was used for final check of the prepared libraries. 16S rRNA sequencing was performed in Illumina Miseq platform after spiking the denatured pools with PhiX DNA (10%) with MiSeq V3 reagent kit.

#### Amplification of ITS Region with PCR

DNA isolated from cecal samples from the experimental groups and DNA isolated from pooled infant stools were screened for fungi. ITS5 (forward, GGAAGTAAAAGTCGTAACAAGG) and ITS4 (reverse, TCCTCCGCTTATTGATATGC) primers were used to amplify the internal transcribed region including the 5.8 ribosomal gene (19). PCRs were performed in 25 µl volume using 0.65 U Dream Taq DNA polymerase (Thermo Fisher Scientific), 0.20 µM primers, 200 µM dNTP mix, and 2 mM MgCl_2_. The thermal program was the following: denaturation at 95°C for 5 min, 35 cycles of 95°C for 30 s, 55°C for 30 s, 72°C for 60 s, and final elongation at 72°C for 7 min. 25 ng of the templates was used. *Candida albicans* DNA (isolate 016, a kind gift from Prof. Per Ljungdahl) was used as positive control, as well as C57BL/6 mouse DNA as negative control. To verify the amplicons, 5 µl of the PCR mixes were loaded on a 1.5% agarose gel stained with GelRed (Biotium).

#### Sequencing of ITS Region

ITS5 (forward, GGAAGTAAAAGTCGTAACAAGG) and ITS4 (reverse, TCCTCCGCTTATTGATATGC) primers were used to amplify the internal transcribed region including the 5.8 ribosomal gene (19). PCR reactions were performed in 50 µl volume using 1 U Phusion high-fidelity DNA polymerase (Thermo Fisher Scientific), 0.20 µM primers, 200 µM dNTP mix, and 2.5 mM MgCl_2_. The thermal program was the following: denaturation at 98°C for 30 s, 30 cycles of 98°C for 10 s, 55°C for 15 s, 72°C for 30 s, and final elongation at 72°C for 5 min. To verify the amplicon size, 5 µl of the PCR mixes were loaded on a 1.5% agarose gel stained with GelRed (Biotium). PCR products were purified using AMPure XP Beads (Beckman Coulter) according to the manufacturer’s protocol. Concentrations were estimated using Qubit Broad Range ds DNA reagents (Invitrogen). Due to low-DNA concentrations, 10 samples were excluded from further procedures. Eight samples were pooled in equimolar amounts and preceded for adapter ligation using TruSeq DNA PCR-free LT Library Preparation Kit (Illumina). PCR product from *C. albicans* strain was proceeded separately and used as positive control in sequencing. Sequencing was performed in Illumina Miseq platform after spiking the denatured pools with PhiX DNA (5%) with MiSeq V3 reagent kit.

#### Bioinformatics and Statistical Analysis

Every read-pair was merged using Pear 9.10 (20) and then demultiplexed using our own developed script (unpublished). Reads with two recognizable barcodes were sorted into each sample. Quality filtering, dereplication, clustering, and taxonomical application were performed through the Uparse pipeline (https://www.ncbi.nlm.nih.gov/pubmed/23955772). Briefly, sequences with an expected error threshold of 3 and a minimum length of 275 bp passed quality filtering. For OTU clustering, dereplication was done to remove identical sequences and improve the clustering time. Then, the sequences in the FASTA file were sorted in order from the most frequent to the least frequent and singletons were removed by default. Usearch uses a heuristic clustering algorithm with 98.5% identity or radius of 1.5%. Taxonomic classification of each OTU was carried out using the SINA aligner against the SILVA database. The 16S rRNA sequence data have been deposited in the NCBI BioProject with accession number PRJNA382773.

## Results

### Human Infant Microbiota Associated With Allergy Promotes Small Intestinal Th17 Responses

**Figure 1:**
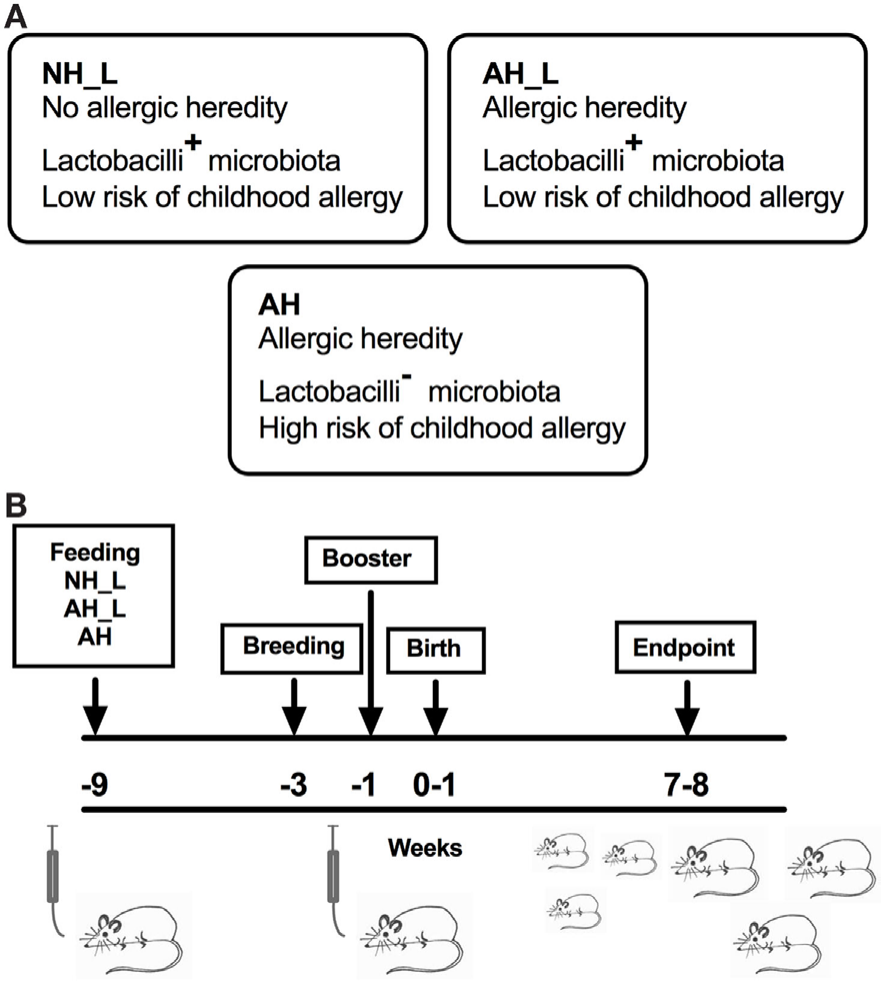
Human microbiota-associated mouse model. Germ-free (GF) mice were inoculated with human infant microbiota, allowed to breed and the offspring was subsequently characterized. **(A)** Human infant stool, collected between 2 and 8 weeks after birth, was pooled from six to seven children per group from the following groups. no allergic heredity, lactobacilli+ (NH_L), allergic heredity, lactobacilli+ (AH_L), and allergic heredity, lactobacilli− (AH). The NH_L and AH_L pooled material was associated with low risk of childhood allergy. The AH stool was collected from children who all became allergic during childhood. **(B)** Overview of the colonization protocol. Male and female GF mice were inoculated with human infant microbiota (*t* = −9 weeks), mated (*t* = −3 weeks), and the females were given a booster inoculum (*t* = −1 week) before birth of the offspring (*t* = 0–1 week). The offspring was culled and analyzed at 7–8 weeks of age.

In order to investigate the links between early-life microbial exposure of the intestinal tract and subsequent immune system phenotype, the effects of different types of human microbiota were tested by transplanting infant microbiota into GF mice and examining the offspring of these mice. We used fecal samples collected from children aged 2–8 weeks who were part of the previously described cohort (14, 16). These children were all healthy and born full-term. To avoid factors known to affect the composition of the microbiota, the children had all been vaginally delivered, were exclusively breastfed and had not been treated with antibiotics during the time of sampling. Microbiota NH_L and AH_L were pooled stools sampled from children with NH or AH, respectively, and with a known presence of group I lactobacilli (L) (Figure 1A). These children were at low risk of exhibiting childhood allergy. Microbiota AH was pooled stool from children with AH but with no trace of group I lactobacilli (Figure 1A). These children all developed childhood allergy. GF mice were fed the differently sourced pooled infant stools by oral gavage. The immune responses in the offspring of the formerly GF mice were subsequently studied when the animals had reached 7–8 weeks of age (Figure 1B). As part of the general characterization, mice were weighed at euthanasia (Figure S1A in Supplementary Material), and although the AH mice as a group displayed significantly lower weight than the AH_L mouse group, no differences in proportions of fat to lean mass as measured by MRI technique were observed between groups (Figure S1B in Supplementary Material).

**Figure 2:**
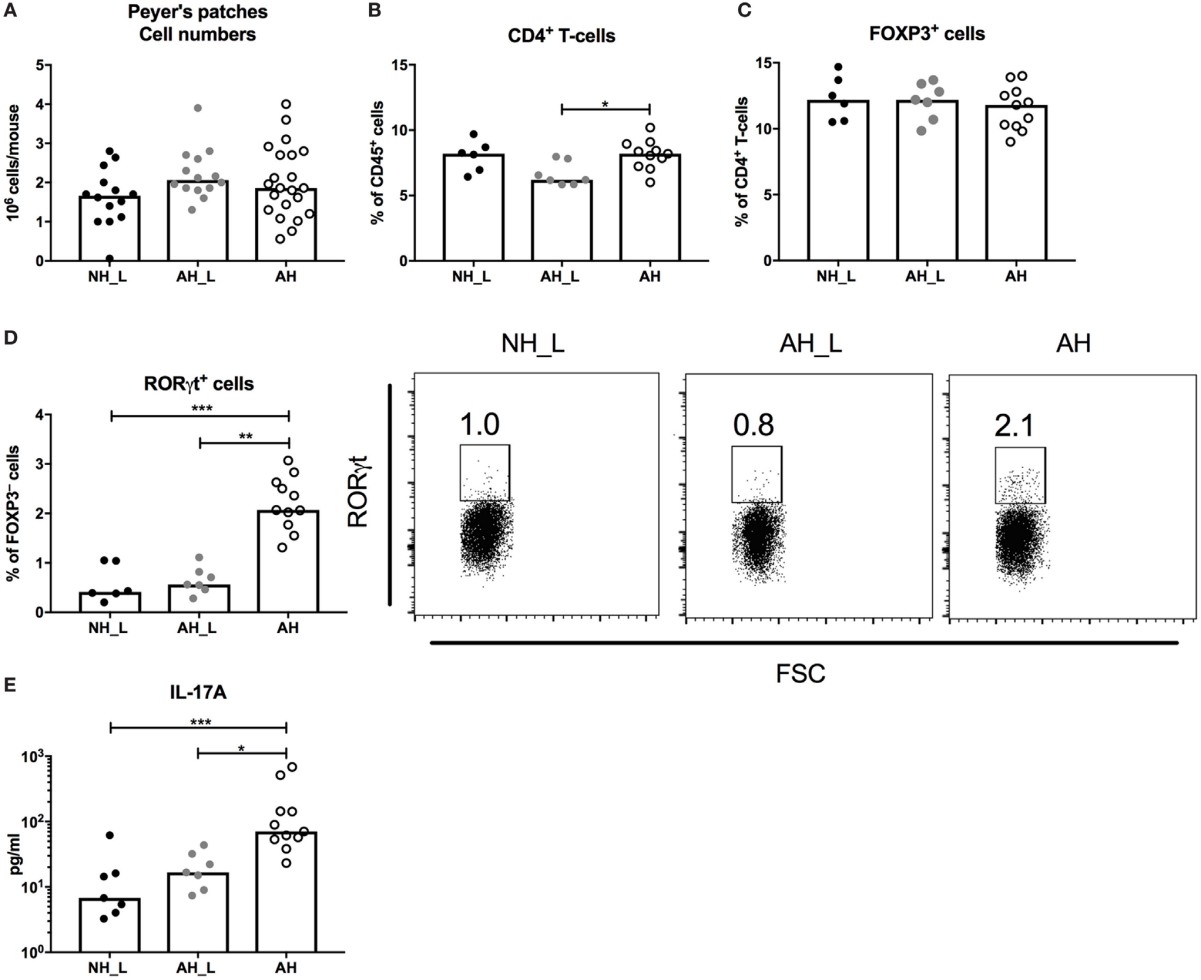
CD4^+^ T-cell responses in the small intestinal mucosa. Peyer’s patches (PP) lymph nodes were isolated from small intestinal tissue, processed, and the single cells were further characterized. **(A)** Total cell numbers in isolated PP. Each symbol is equivalent to one individual animal [*n* = 14 no allergic heredity, lactobacilli+ (NH_L), *n* = 14 allergic heredity, lactobacilli+ (AH_L), and *n* = 22 AH]. **(B)** Percentages of CD4^+^ T-cells within the CD45^+^ population in PP. **(C)** Proportions of FOXP3^+^ cells within the CD4^+^ T-cell population. **(D)** Proportions of RORγt^+^ cells within the FOXP3^−^CD4^+^ T-cell population. **(E)** Mucosal IL-17A in culture supernatants following stimulation of single cells from PP with αCD3/αCD28 for 24 h. For **(B–E)**, each symbol is equivalent to pooled material from two mice. Bars show median values. For statistical analysis, one-way ANOVA Kruskal–Wallis test with Dunn’s multiple comparisons test was used. *p < 0.05, **p < 0.01, ***p < 0.001.

The PP are major sites for the induction of intestinal immune responses (21). Initial characterization of the PP revealed no differences in total cell numbers (Figure 2A) and all groups exhibited similar proportions of B-cells and total T-cells (Figures S2A,B in Supplementary Material). The AH_L mice had reduced proportions of CD4^+^ T-cells (Figure 2B), but no differences in the proportion of FOXP3^+^CD4^+^ T-cells were detected between the groups (Figure 2C). However, when investigating the potential development of effector T-cells in the FOXP3^–^CD4^+^ T-cell population, a distinct increase of RORγt^+^ T-cells in the AH group was observed (Figure 2D), as well as significantly increased production of IL-17A by PP cells stimulated with αCD3 and αCD28 (Figure 2E). The complete gating strategy is displayed in (Figure S3 in Supplementary Material). Together these findings strongly indicated the induction of intestinal Th17-cells in animals exposed to the AH microbiota.

### RORγt Expression in Colonic CD4^+^ T-Cells Is Highly Promoted by Allergy-Associated Microbiota

**Figure 3:**
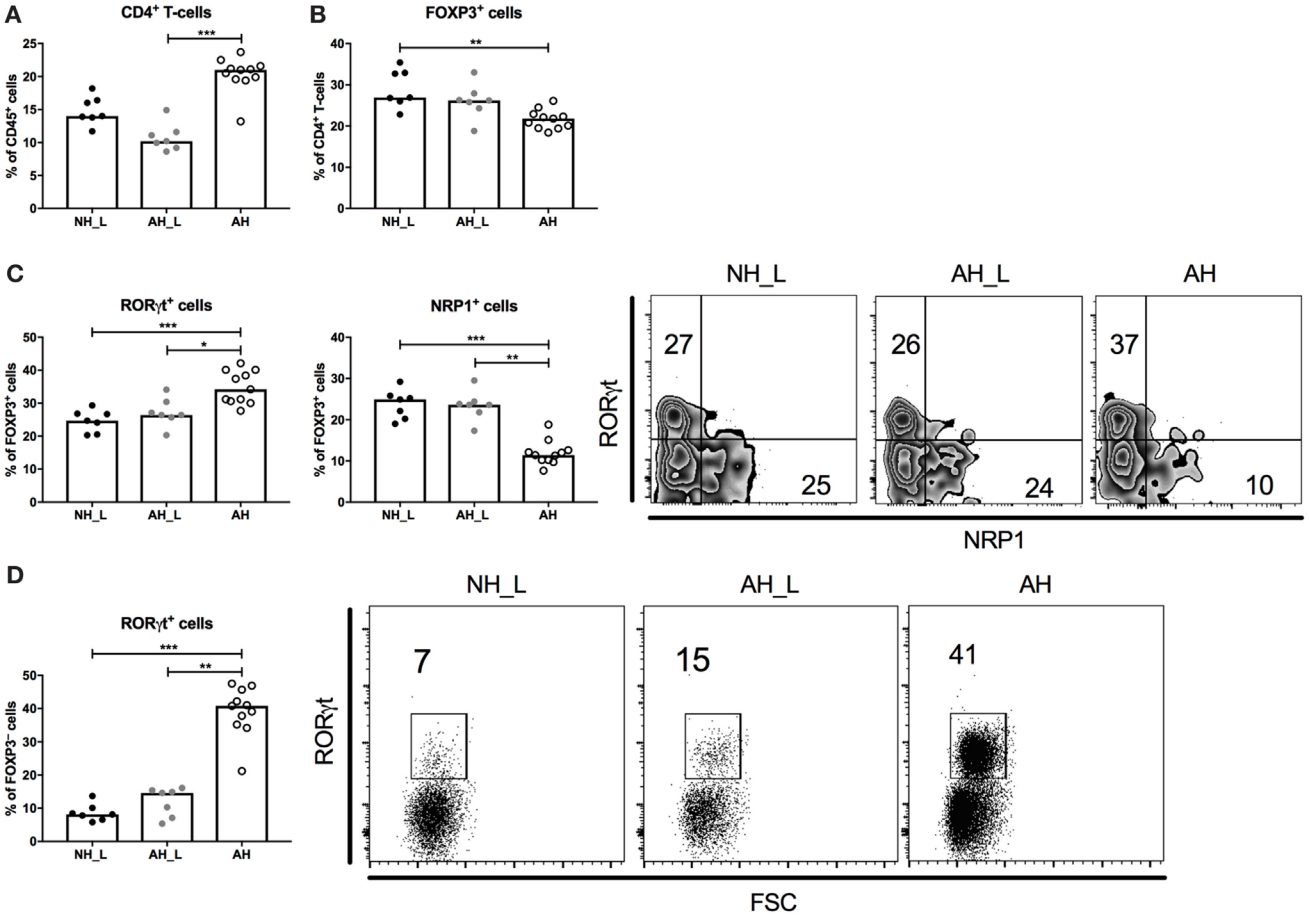
Characterization of the CD4^+^ T-cell compartment in the colonic lamina propria. Lamina propria leukocytes (LPL) were isolated from colonic tissue and further characterized. **(A)** Percentages of CD4^+^ T-cells within the CD45^+^ population in LPL. **(B)** Proportions of FOXP3^+^ cells within the CD4^+^ T-cell population. **(C)** Proportions of RORγt^+^ and NRP1^+^ cells within the FOXP3^+^CD4^+^ T-cell compartment. **(D)** Proportions of RORγt^+^ cells within the FOXP3^−^CD4^+^ T-cell compartment. Each symbol is equivalent to pooled material from two individual mice [*n* = 7 no allergic heredity, lactobacilli+ (NH_L), *n* = 7 allergic heredity, lactobacilli+ (AH_L), and *n* = 11 AH]. Bars represent median values. For statistical analysis, one-way ANOVA Kruskal–Wallis test with Dunn’s multiple comparisons test was performed. *p < 0.05, **p < 0.01, ***p < 0.001.

We further investigated CD4^+^ T-cell responses in the colon, for which the complete gating strategy is displayed in Figure S4 in Supplementary Material. Initial characterization of the cLP T-cells showed that the total CD4^+^ T-cell population was increased in the AH group (Figure 3A), although the proportion of FOXP3^+^CD4^+^ T-cells was slightly reduced in this group (Figure 3B). When examining the FOXP3^+^ cell population further, the AH cohort emerged with a distinct shift to RORγt-expressing cells (Figure 3C), indicative of regulatory T-cells (Treg) that had been induced in the periphery after microbial exposure (22–24). NRP1-expressing cells, regarded as thymic-derived Treg (25), constituted a smaller fraction of the total FOXP3^+^CD4^+^ T-cell population in these mice (Figure 3C). Analysis of the FOXP3^−^ compartment of CD4^+^ T-cells revealed that similarly to the PP, RORγt expression was markedly increased in the AH group compared with both the NH_L and the AH_L groups (Figure 3D). Overall, the AH microbiota provided robust cues for CD4^+^ T-cell accumulation and RORγt expression in the gut.

### Systemic Th17 Responses Are Promoted by Allergy-Associated Microbiota

**Figure 4:**
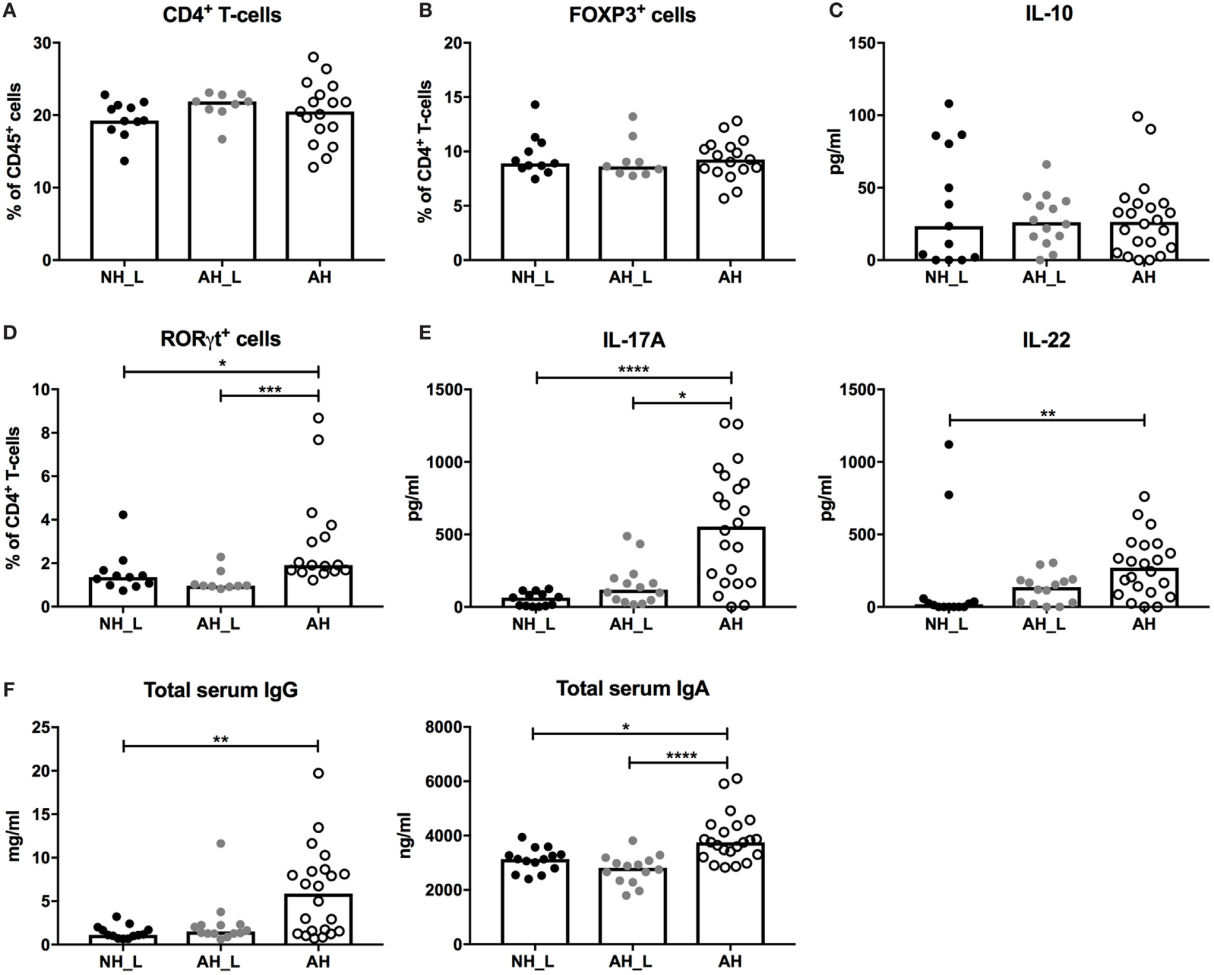
Systemic CD4^+^ T-cell responses. Systemic T-cell responses were evaluated by characterizing isolated splenocytes. **(A)** Percentages of CD4^+^ T-cells within the CD45^+^ population in spleen. **(B)** Proportions of FOXP3^+^ cells within the CD4^+^ T-cell population. Each symbol is equivalent to one individual animal **(A,B)**: *n* = 11 no allergic heredity, lactobacilli+ (NH_L), *n* = 9 allergic heredity, lactobacilli+ (AH_L), and *n* = 17 AH. **(C)** Systemic IL-10 in culture supernatants following stimulation of single cells from spleen with αCD3/αCD28 for 24 h (*n* = 13 NH_L, *n* = 14 AH_L, and *n* = 22 AH). **(D)** Proportions of RORγt^+^ cells within the CD4^+^ T-cell compartment (*n* = 11 NH_L, *n* = 9 AH_L, and *n* = 16 AH). **(E)** Systemic IL-17A and IL-22 in culture supernatants following stimulation of single cells from spleen with αCD3/αCD28 for 24 h (*n* = 13 NH_L, *n* = 14 AH_L, and *n* = 22 AH). **(F)** Levels of total IgG and IgA in serum (*n* = 13 NH_L, *n* = 14 AH_L, and *n* = 22 AH). Bars represent median values. For statistical analysis, one-way ANOVA Kruskal–Wallis test with Dunn’s multiple comparisons test was performed. *p < 0.05, **p < 0.01, ***p < 0.001, ****p < 0.0001.

We further examined systemic T-cell populations and responses by isolating cells from the spleen. The AH mice displayed a significantly increased percentage of CD3^+^ T-cells compared with the NH_L mice (Figure S5A in Supplementary Material), but the increased population was not due to accumulation of CD4^+^ T-cells (Figure 4A). Furthermore, no differences in the percentage FOXP3^+^ T-cells (Figure 4B), or in αCD3/αCD28-induced secretion of IL-10 from splenocytes (Figure 4C) were observed. In order to investigate general Th1 and Th2 responses, splenocyte production of IFNγ and IL-4 following stimulation with αCD3/αCD28 was examined, but no significant differences were noted between groups (Figure S5B in Supplementary Material).

In line with the gut compartments, the proportion of RORγt-expressing CD4^+^ T-cells was significantly increased in the AH group (Figure 4D). Also, splenocytes isolated from AH mice produced significantly more IL-17A following αCD3/αCD28 stimulation (Figure 4E), revealing increased Th17 responses. In addition, IL-22, which can be produced by Th17-cells (26), was increased in stimulated splenocytes from the AH group (Figure 4E). Production of innate cytokines IL-6 and IL-1β, which are needed for development of functional Th17-cells (27, 28), was similar in the experimental groups (Figure S5C in Supplementary Material). The level of IL-23, which drives IL-22 expression by Th17 (29), was slightly increased in the AH group (Figure S5C in Supplementary Material).

B-cell responses, such as IgE production, are also affected by microbial context (30), which could have an impact on later life establishment of allergy. Levels of serum IgG and serum IgA were significantly elevated in the AH group (Figure 4F), indicating that increased B-cell class switching had occurred. Percentages of total B-cells, IgM^+^ B-cells and IgA^+^ B-cells were equivalent between groups (Figures S6A–C in Supplementary Material). In addition, no differences in the proportion of marginal zone B-cells, which are known producers of natural antibodies directed at the commensal microbiota (31), could be detected (Figure S6D in Supplementary Material). The complete gating strategy for B-cells is displayed in (Figure S7 in Supplementary Material). The elevated serum antibody levels show that AH mice displayed altered functional B-cell properties; however, this was not reflected in changes in the splenic cellular B-cell compartment.

### Allergy-Associated Microbiota Does Not Induce Intestinal Inflammation

**Figure 5:**
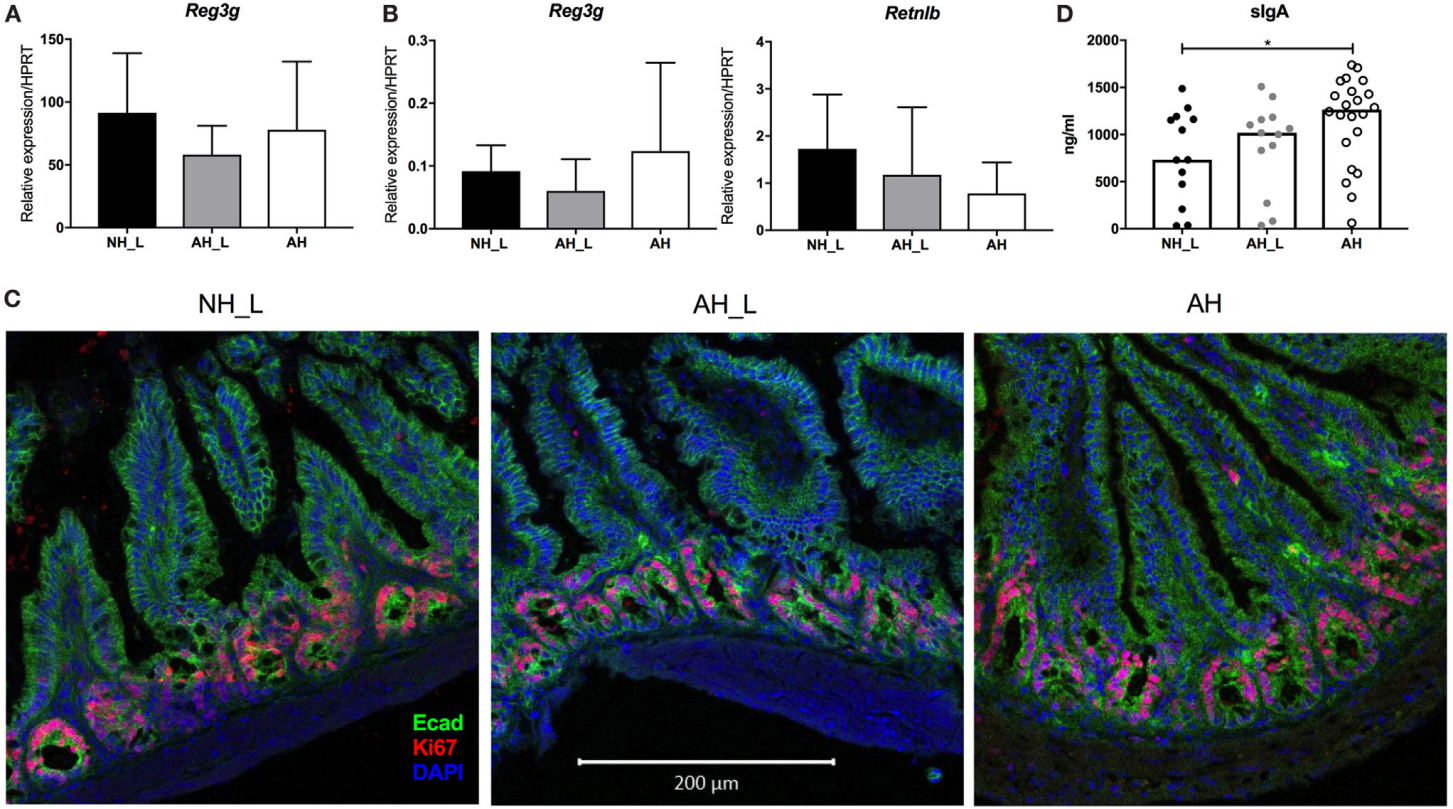
Barrier properties of the intestinal mucosa. Gene expression and cellular proliferation were examined in intestinal tissue. **(A)** mRNA expression of the antimicrobial peptide (AMP) *Reg3g* in small intestinal tissue. **(B)** mRNA expression levels of the AMPs *Reg3g* and *Retnlb* in colonic tissue. For **(A,B)**, *n* = 8 mice were analyzed per experimental group, and data displayed as mean ± SD. **(C)** Intestinal epithelial cell proliferation in distal small intestine. Representative small intestinal sections from no allergic heredity, lactobacilli+ (NH_L), allergic heredity, lactobacilli+ (AH_L), and AH mice were stained with E-cadherin, Ki67, and DAPI. Images were acquired with the 20× objective. The white bar is equivalent to 200 µm. **(D)** Levels of secretory IgA in small intestinal lumen. Total IgA was measured in small intestinal content using enzyme-linked immunosorbent assay. Each symbol is equivalent to an individual mouse (*n* = 13 NH_L, *n* = 13 AH_L, and *n* = 22 AH) and bars represent median values. For statistical analysis, one-way ANOVA Kruskal–Wallis test with Dunn’s multiple comparisons test was performed. *p < 0.05.

Considering that the AH mice exhibited both increased intestinal and systemic Th17 responses, this could indicate that they harbored more reactive or pathogenic microbiota. The intestinal mucosa was therefore examined for alterations in barrier properties or the presence of obvious inflammation. Antimicrobial peptides (AMP) play an important role in protecting the intestinal epithelium. The C-type lectin REGIIIγ is known to exist in high concentrations in the small intestine, where it is produced by IEC and contributes to maintaining the barrier (32). All experimental groups displayed equivalent *Reg3g* mRNA expression levels in small intestinal tissue (Figure 5A), excluding differential effects of particular microbes on a major AMP. In the colon tissue, only low levels of *Reg3g* could be detected (Figure 5B). Further, no differences in expression of the AMP *Retnlb*, which is constitutively expressed by the colonic epithelium (33), could be observed (Figure 5B).

Altered epithelial cell turnover could be a sign of acute infection with intestinal pathogens or of chronic inflammation of the gut. IEC proliferation was determined in small intestinal tissue by immunofluorescence staining of Ki67. Actively proliferating IEC were similarly localized in the crypts in all experimental groups (Figure 5C), indicating that the Th17-skewed response observed both locally and systemically, was not secondary to intestinal disease. Furthermore, myeloid immune cell frequencies in PP were similar between the experimental groups, as was the composition of intraepithelial lymphocytes in small intestinal tissue (data not shown). However, secretory IgA, which is induced during colonization with intestinal bacteria (34), was significantly elevated in the AH group compared with the NH_L animals (Figure 5D), again indicating that AH microbiota was associated with more robust mucosal immune responses.

### Allergy-Associated Microbiota Has a Distinct Microbial Profile

**Figure 6:**
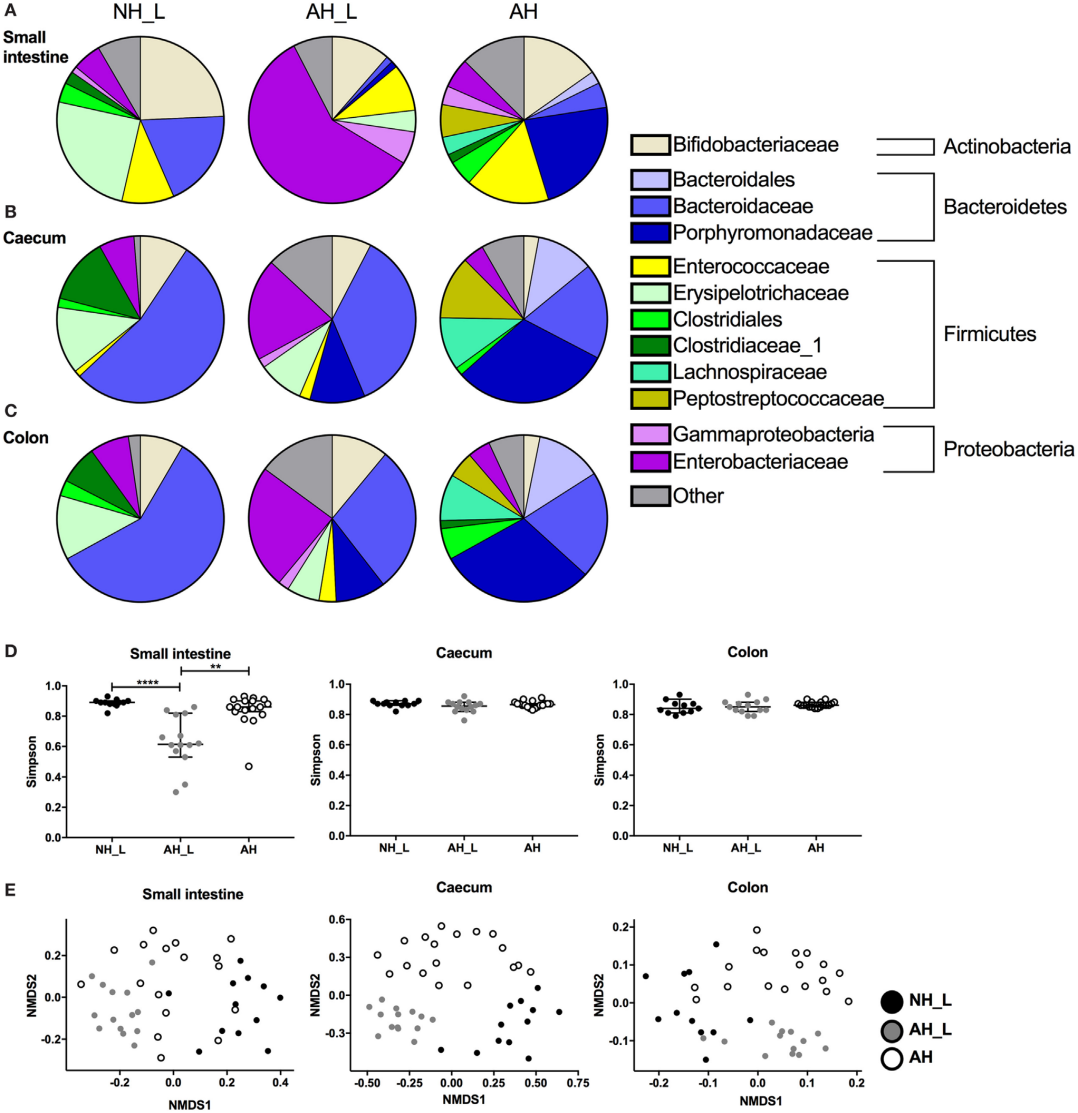
Properties of microbial communities across the gastrointestinal tract. The microbial communities in small intestine, cecum, and colon were analyzed. Intestinal contents were collected, DNA extracted, and characterization of the bacterial communities performed using Illumina 16S rRNA sequencing. **(A–C)** OTU relative abundance of combined data for each cohort in **(A)** small intestine, **(B)** cecum, and **(C)** colon. **(D)** Simpson’s diversity index was determined for sequencing data from the small intestine, cecum, and colon. Data are displayed with median values and 95% confidence interval. **(E)** Non-metric multidimensional scaling analysis was used to determine dissimilarities of bacterial communities within the different intestinal loci. For **(D,E)**, each symbol is equivalent to an individual animal (small intestine *n* = 12 no allergic heredity, lactobacilli+ (NH_L), *n* = 14 allergic heredity, lactobacilli+ (AH_L), *n* = 17 AH, cecum *n* = 12 NH_L, *n* = 14 AH_L, *n* = 18 AH, and colon *n* = 11 NH_L, *n* = 13 AH_L, *n* = 18 AH). For statistical analysis one-way ANOVA Kruskal–Wallis test with Dunn’s multiple comparisons test was used. **p < 0.01, ****p < 0.0001.

Although the cecum and colon harbor the highest loads of bacteria in the intestinal tract, the small intestine is an important site for the induction of gut immune responses. In order to obtain a comprehensive view of gut microbiota composition in the experimental groups, content from small intestine, cecum and colon were collected from individual animals at dissection and their microbiome composition analyzed separately using 16S rRNA sequencing. The major phyla detected throughout the intestinal tract were Actinobacteria, Bacteroidetes, Firmicutes, and Proteobacteria (Figures 6A–C), which was in line with a previous study of human gastrointestinal microbiota (35).

Overall, the small intestine displayed a distinct microbial profile compared with the cecal and colonic compartments, which were similar within the respective groups. Bifidobacteriaceae, a group of bacteria known to metabolize human milk oligosaccharides (36), were ubiquitous, although the relative abundance was higher in NH_L mice compared with AH mice in all three sampled sites (Figures 6A–C). In the small intestine, the Erysipelotrichaceae were a characteristic of the NH_L group (Figure 6A) and could only be detected at very low abundance in the AH animals (Table S1 in Supplementary Material). Similarly, the Porphyromonadaceae were typical of the AH group (Figure 6A) but were only present at low-relative abundance in NH_L mice (Table S1 in Supplementary Material). AH_L animals differed markedly from both NH_L and AH animals in all three sampled sites. In the small intestine, the Enterobacteriaceae dominated (Figure 6A) and AH_L mice exhibited a lower diversity index at this site, while diversity remained high in both the NH_L and AH mice at all sampled loci (Figure 6D).

In both the NH_L and AH cohorts, the cecal and colonic microbiota were dominated by Bacteroidetes, more specifically by Bacteroidaceae in the NH_L mice and by Porphyromonadaceae in AH mice (Figures 6B,C). Firmicutes constituted the second most abundant phylum in the lower intestinal tract and were mainly comprised of Erysipelotrichaceae and Clostridiaceae in the NH_L mice (Figures 6B,C). Although AH mice harbored Clostridiaceae in the colon, the relative abundance was low and two other members of Clostridiales, Lachnospiraceae, and Peptostreptococcaceae were the most prevalent Firmicutes in both cecum and colon of this group (Figures 6B,C). The AH_L group exhibited traits associated with both NH_L and AH animals, in that both Erysipelotrichaceae and Porphyromonadaceae were present (Figures 6B,C). Contrary to both the NH_L and AH cohorts, only smaller proportions of Firmicutes could be detected in AH_L mice and members of Clostridiales were largely absent (Figures 6A–C; Table S1 in Supplementary Material). Non-metric multidimensional scaling analysis of microbiota revealed that the dissimilarities within the groups were particularly small in the AH_L group (Figure 6E).

The human commensal fungus *C. albicans* has been shown to induce Th17 responses *via* activation of the innate immune system (37). Therefore, the presence of fungi was examined in the experimental groups by PCR screening of DNA extracted from cecal content using general fungal ITS primers. The results revealed fungal presence in the AH group of mice as evidenced by the positive signal for this 18S ITS (Figure S8 in Supplementary Material). Sequencing of the ITS region identified reads that were confirmed to be *C. albicans* in the AH mice (data deposited in the NCBI BioProject with accession number PRJNA382773).

## Discussion

This study shows that when human infant microbiota associated with both AH and prospective allergy was used to colonize GF mice, it resulted in offspring with increased intestinal and systemic Th17 responses, characterized by increased RORγt expression among CD4^+^ T-cells, and FOXP3^−^CD4^+^ T-cells in particular, as well as increased IL-17A secretion. RORγt^+^CD4^+^ T-cells are found in various organs and tissues, but particularly accumulate in the intestinal mucosa (38).

The small intestine is known to be an important site for induction of Th17 responses (39). In our study, we observed a clear Th17 induction in the small intestine of the AH mice only, with a T-cell dependent production of IL-17A and the emergence of RORγt^+^FOXP3^−^CD4^+^ T-cells. We also detected elevated secretory IgA in the small intestine of AH mice, which further indicated Th17 induction, since Th17-cells are closely linked to mucosal IgA production (40). This is in line with studies where specific colonization with segmented filamentous bacteria (SFB), which are known to induce specifically Th17 cell development in the small intestinal mucosa (41, 42), has been associated with increased expression of IgA in the epithelial barrier (43).

The population of RORγt^+^FOXP3^−^CD4^+^ T-cells in the cLP of AH mice was significantly expanded compared with the other groups. A proportion could be Th17-cells, which are present in the colonic mucosa of conventional mice (44). Regardless of cytokine profile, one study showed that colonic RORγt^+^FOXP3^−^CD4^+^ T-cells likely are part of general responses to established intestinal microbes (24), if the bacteria provide the correct stimulus. The AH microbiota was capable of inducing such immune development, which made it unique compared with NH_L and AH_L microbiota. Increased frequencies of RORγt-expressing colonic FOXP3^+^CD4^+^ T-cells, which are indicative of peripheral Tregs, further supported a state of increased microbial stimulation in AH mice. RORγt^+^ Tregs have been shown to exhibit strong suppressive characteristics and emerge following colonization with bacteria during post-natal development (22–24). The increased expression of RORγt by CD4^+^ T-cells in AH mice could indicate that accelerated bacterial colonization and subsequent immune maturation had occurred in these mice during infancy.

It is important to acknowledge that other immune cells contribute to cytokine-production in the gut, besides CD4^+^ T-cells. Innate lymphoid cells are an important part of the innate immune system in the gut and are known to modulate immune responses to the intestinal microbiota. They respond to factors produced by other cell types in the gut, such as myeloid cells, and produce cytokines, among them IL-17A (45).

In addition to elevated local Th17 responses, a Th17 signature was present in systemic CD4^+^ T-cells in AH mice. The gut microbiota is known to have an impact on immune responses at other sites in the body, especially the lungs. This has been described as the gut-lung axis (46). Established colonization with SFB as part of a complex microbiota was reported as beneficial in a model of bacterial pneumonia, resulting in reduced infectious burden and disease. The protective function was mediated by IL-22, most likely produced by Th17- or Th22-cells (47). Importantly, however, properties that are advantageous during acute infection might be detrimental in chronic inflammatory settings. Manipulation of intestinal microbiota in murine models of allergic asthma (48) and hypersensitivity pneumonitis (49), resulting in variable degrees of disease severity, revealed that amelioration was coupled to reduced mixed Th1/Th17 responses in lung tissue. Although we did not evaluate the immune response in lung tissues in this study, the increased population of gut mucosal and systemic Th17-cells that we observe here might still contribute to an environment prone to increased immune activation, sensitization and allergy, in agreement with the postulated gut-lung axis (46).

Studies have shown that specific bacteria, such as SFB in mice, are capable of inducing Th17 in the intestine. So far, no human equivalent to SFB has been identified. Mono-colonization of GF mice with single species of human commensal bacteria revealed that several species including *Enterococcus faecalis, Staphylococcus saprophyticus*, and *Bifidobacterium adolescentis* induced Th17 in the small intestinal lamina propria to the same degree as SFB. It was further demonstrated that *B. adolescentis* promoted the emergence of RORγt^+^FOXP3^−^CD4^+^ T-cells in the cLP to the same extent as SFB (50). It should be noted, that these species induced different and distinct epigenetic changes in the responding cells, highlighting the complexity of microbiota-induced immune modulation. However, intestinal immune activation is likely induced by a variety of different bacteria. Although a reduced diversity index has generally been regarded as a trait of allergic diseases, we did not detect any differences in microbial diversity between the groups least and most associated with allergy, which was consistent with a previous study of microbiota and prospective allergy (48). Still, the AH_L group did display reduced diversity in the small intestine, which was dominated by Enterobacteriaceae, a group of bacteria known as early gut colonizers in humans (51), which could be a reflection of more immature microbiota. Still this microbiota was associated with low risks of allergy in the children. In terms of immune responses, AH_L mice in some cases formed an intermediary group, but were in general more similar to NH_L animals, suggesting that the Enterobacteriaceae did not have definitive effects on immune development.

This study revealed large differences in the abundance of particularly the bacterial families Erysipelotrichaceae, typical of NH_L mice, and Porphyromonadaceae, a characteristic of AH animals. The Erysipelotrichi class has been identified as a general component of the human microbiota (52) and species within this group were reduced following sensitization in a murine model of experimental food allergy (53). Contrary to this, the same study revealed that species within Porphyromonadaceae were reported as expanded following sensitization (53). Although these bacteria are not extensively described, it is possible that they are indicative of shifts in overall bacterial communities.

Previous research of associations between early-life microbiota and the risks of developing allergy has identified particular microbes or configurations of microbes as associated with prospective Th2-mediated disease (48, 54). Neonatal gut microbiota containing less of certain bacteria, among them Bifidobacteriaceae and Lactobacillaceae, and higher abundance of the fungi *Candida* and *Rhodotorula*, was correlated with the highest risk of atopy and asthma later in childhood, although both fungi were identified as general features of infant microbiota (54). Contrary to this assertion, this study identified fungi only in mice colonized with microbiota associated with prospective allergy, suggesting that fungi could contribute to the Th17-signature observed in the AH mice. A study of the murine gut showed that commensal fungi colonized the same niche as bacteria and that they were particularly abundant in the lower bowel (55), which is the location we chose to analyze for fungal presence. An antagonistic relationship between *C. albicans* and lactobacilli species in the mucosa has been reported from mouse studies (56, 57). For example, lactobacilli contributed to increased resistance to fungal colonization *via* induction of IL-22 (57) which is known to modulate the microbiota (58). In our study, we could detect Lactobacillaceae only in groups NH_L and AH_L (Table S1 in Supplementary Material), which was in agreement with the initial fecal inoculates. Since only a small percentage of sequences in group NH_L and AH_L were derived from Lactobacillaceae, we cannot be sure of how much of a direct impact lactobacilli have on immune responses in the mice. However, we view lactobacilli as signature species of a type of microbiota associated with reduced risk of childhood allergy. Although increased IL-17A production and fungal presence were correlated in AH mice, the impact of bacterial microbiota on Th-cell skewing cannot be dismissed. The slight increase in Th17 responses in AH_L mice, which did not harbor fungi, suggests that bacterial stimuli also contributed to Th17 induction.

Murine models are fundamental for investigating complex immune responses to the intestinal microbiota, but it is important to consider the differences in physiology and immune maturation between mice and humans. The infant mouse is born with a more immature intestinal barrier than human children (59), which will result in different interactions between immune cells and early gut microbiota. Although the majority of human commensal bacteria can be established in mice (52), the relative compositions are altered (60), limiting the translational aspects of our findings to some extent. The use of pooled donors and a full microbiota for the fecal inoculates could be seen as a strength of our study. Instead of overemphasizing features of the microbiota from a single child or single bacterial species, we were interested in general microbiota patterns in groups that share traits. The different pooled infant microbiota samples were also sequenced and we are currently investigating the fidelity of transfer. Further, we performed a pilot study, with slight differences in the inoculation protocol and the immunological analysis, which also showed that only the AH microbiota promoted IL-17A responses, both in the gut and in the systemic compartments, thereby being a partial replicate for the current study. For further increased robustness of our study, we analyzed microbiota and immune responses at several loci in the gastrointestinal tract. Inoculation of GF mice with human microbiota has been reported to result in smaller CD3^+^ T-cell populations in the gut and reduced RORγt expression in T-cells (60). The increased Th17 responses observed in the AH group indicate a more stimulatory type of microbiota, potentially because of the presence of certain microbial features known to induce Th17, such as adhesion to IEC (43, 61).

The number of people suffering from allergies is increasing across the world and asthma alone is expected to affect 400 million people by 2025 (62). The role for Th17 responses in asthma and allergy is becoming increasingly clear (63, 64). Indeed, children with allergic asthma are reported to have increased frequencies of Th17-cells in their circulation (65) and experimental allergy in mice is ameliorated by RORγt-inhibition (66). In our study, we present for the first time an association between allergy-associated microbiota and a clear Th17 signature in multiple compartments. AH is strongly connected with allergy, but the risks of developing disease might be further influenced by the gut microbiota composition. In the light of our findings, mucosal Th17 responses occurring during immune development, and the subsequent effects on sensitization and allergy, should be taken into consideration in allergy research and development of future therapies.

## Author Contributions

DP coordinated immunological experiments and drafted methods. SN wrote the manuscript with input from all authors. DP, SN, KQ, and CC-Q designed immunological experiments, collected, and analyzed data. OA was responsible for the bioinformatics analysis and drafted methods. EH, DLH, and DH performed 16S rRNA sequencing. EH was responsible for fungal ITS sequencing and drafted methods. SB provided assistance with the experimental analyses and interpretation of data. YH, MN, IL, EK, JH, and BB provided experimental assistance. CN performed clinical examinations and allergy diagnosis of the children. KU planned and supervised the sequencing. ES-E envisioned and designed the overall study.

## Conflict of Interest Statement

The authors declare that the research was conducted in the absence of any commercial or financial relationships that could be construed as a potential conflict of interest.

## Abbreviations

AH, allergic heredity; AH_L, allergic heredity, lactobacilli+; AMP, antimicrobial peptide; cLP, colonic lamina propria; ELISA, enzyme-linked immunosorbent assay; GF, germ-free; IEC, intestinal epithelial cells; Ig, immunoglobulin; NH_L, no allergic heredity, lactobacilli+; PBS, phosphate-buffered saline; PP, Peyer’s patches; Th, T helper; Treg, regulatory T-cells.
