# Supplementary material for: Early-Life Human Microbiota Associated With Childhood Allergy Promotes the T Helper 17 Axis in Mice
Source: Front Immunol. 2017 Dec 1;8:1699. doi: 10.3389/fimmu.2017.01699 (PMC5716970; doi:10.3389/fimmu.2017.01699)
Supplement: Supplementary file 1 [file Table_1.pdf]

**Supplementary Table 1.** Microbial composition of the category designated as Other. General Bacteria, unclassified sequences and known entities of relative abundance <1.2%. Taxa replaced with * constitute a relative abundance ≥1.2% and are represented in Figures 6A, B and C.

| Small intestinal content | NH_L | AH_L | AH |
|---|---|---|---|
| Actinomycetaceae | 0.00000000% | 0.00000000% | 0.49226800% |
| Bacteria | 0.04930970% | 2.72310278% | 2.34697323% |
| Bacteroidales | 0.00000000% | 0.01207584% | * |
| Chromatiaceae | 0.05635390% | 0.00000000% | 0.28342700% |
| Clostridiaceae_1 | * | 0.01811380% | * |
| Clostridiales | * | 0.00603792% | * |
| Comamonadaceae | 0.31699100% | 0.00000000% | 1.16851000% |
| Coriobacteriaceae | 0.33812300% | 0.00000000% | 1.13371000% |
| Corynebacteriales | 0.00000000% | 0.00000000% | 0.00497240% |
| Erysipelotrichaceae | * | * | 0.10939300% |
| Gammaproteobacteria | * | * | * |
| Lachnospiraceae | 0.06339810% | 0.00000000% | * |
| Lactobacillaceae | 0.00704424% | 0.00000000% | 0.00000000% |
| Microbacteriaceae | 0.00000000% | 0.00000000% | 0.00994481% |
| Moraxellaceae | 0.00000000% | 0.00000000% | 0.00994481% |
| Mycobacteriaceae | 0.01408850% | 0.00000000% | 0.00000000% |
| Paenibacillaceae | 0.00000000% | 0.00000000% | 0.01491720% |
| Peptostreptococcaceae | 0.02113270% | 0.00000000% | * |
| Porphyromonadaceae | 0.20428300% | * | * |
| Propionibacteriaceae | 0.00704424% | 0.00000000% | 0.00000000% |
| Proteobacteria | 0.00704424% | 0.00000000% | 0.00000000% |
| Rhizobium | 0.00000000% | 0.00000000% | 0.00497240% |
| Ruminococcaceae_UCG-002 | 0.00000000% | 0.00000000% | 0.00497240% |
| Sanguibacteraceae | 0.00000000% | 0.00000000% | 0.00497240% |
| Sphingobacteriaceae | 0.00000000% | 0.00000000% | 0.06961360% |
| Staphylococcaceae | 0.00000000% | 0.02415170% | 0.00497240% |

| | | | |
|---|---|---|---|
| Unclassified | 7.31191562% | 4.80618080% | 6.62821421% |
| Xanthomonadaceae | 0.00000000% | 0.00000000% | 0.26851000% |

| Caecal content | NH_L | AH_L | AH |
|---|---|---|---|
| Actinomycetaceae | 0.00000000% | 0.00000000% | 0.05269290% |
| Bacteria | 0.06991940% | 10.63644631% | 4.95820069% |
| Bacteroidales | 0.00000000% | 0.05797666% | * |
| Clostridiaceae_1 | * | 0.06905090% | 0.92212600% |
| Clostridiales | * | 0.00455996% | * |
| Comamonadaceae | 0.00000000% | 0.02345120% | 0.00000000% |
| Coriobacteriaceae | 0.08967930% | 0.00000000% | 0.34706400% |
| Corynebacteriaceae | 0.00000000% | 0.09575920% | 0.00000000% |
| Enterococcaceae | * | * | 0.16719900% |
| Erysipelotrichaceae | * | * | 0.00000000% |
| Gammaproteobacteria | 0.91123225% | * | 0.90641925% |
| Lachnospiraceae | 0.07523940% | 0.10683300% | * |
| Lactobacillaceae | 0.00227998% | 0.00260569% | 0.00000000% |
| Methylobacterium | 0.00000000% | 0.01107420% | 0.00000000% |
| Oxalobacteraceae | 0.00000000% | 0.03126830% | 0.00000000% |
| Peptostreptococcaceae | 0.00000000% | 0.09510780% | * |
| Porphyromonadaceae | 0.00000000% | * | * |
| Propionibacteriaceae | 0.00000000% | 0.02345120% | 0.00000000% |
| Proteobacteria | 0.00000000% | 0.00000000% | 0.00101333% |
| Rhizobiales | 0.00000000% | 0.01758840% | 0.00000000% |
| Sphingomonadaceae | 0.00000000% | 0.00130285% | 0.00000000% |
| Staphylococcaceae | 0.00000000% | 0.00977135% | 0.00000000% |
| Thermaceae | 0.00000000% | 0.04690250% | 0.00000000% |
| Unclassified | 0.10639909% | 1.81877398% | 0.94441835% |

| Colonic content | NH_L | AH_L | AH |
|---|---|---|---|

| | | | |
|---|---|---|---|
| Actinomycetaceae | 0.00000000% | 0.00000000% | 0.05732470% |
| Bacteria | 0.12129841% | 12.80500432% | 4.52074494% |
| Bacteroidales | 0.00485193% | 0.00000000% | * |
| Clostridiaceae_1 | * | 0.01094800% | * |
| Clostridiales | * | 0.00000000% | * |
| Comamonadaceae | 0.00323462% | 0.00136849% | 0.00197671% |
| Coriobacteriaceae | 0.06145780% | 0.00000000% | 0.24807800% |
| Enterococcaceae | 0.98817700% | * | 0.20459000% |
| Erysipelotrichaceae | * | * | 0.00098836% |
| Gammaproteobacteria | 0.05498860% | * | 1.02097000% |
| Lachnospiraceae | 0.15364500% | 0.00000000% | * |
| Obscuribacterales | 0.00000000% | 0.00547398% | 0.00000000% |
| Oxalobacteraceae | 0.00000000% | 0.00136849% | 0.00000000% |
| Peptostreptococcaceae | 0.00323462% | 0.00273699% | * |
| Porphyromonadaceae | 0.37521600% | * | * |
| Propionibacteriaceae | 0.05337130% | 0.00000000% | 0.00000000% |
| Proteobacteria | 0.00000000% | 0.00000000% | 0.00593014% |
| Staphylococcaceae | 0.01293850% | 0.00684247% | 0.00000000% |
| Unclassified | 0.49166224% | 2.07053250% | 0.81638255% |
