# Supplementary material for: Early-Life Human Microbiota Associated With Childhood Allergy Promotes the T Helper 17 Axis in Mice
Source: Front Immunol. 2017 Dec 1;8:1699. doi: 10.3389/fimmu.2017.01699 (PMC5716970; doi:10.3389/fimmu.2017.01699)
Supplement: Supplementary file 2 [file Image_1.pdf]

SUPPLEMENTARY FIGURES

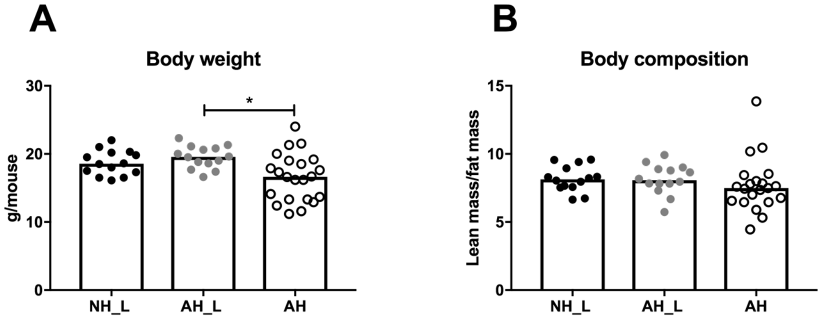

Supplementary Figure 1. Weight and body composition of mice. (**A**) Body weight at endpoint. Each symbol represents an individual mouse (*n*=14 NH_L, *n*=14 AH_L, *n*=22 AH) and bars represent median values. (**B**) Body composition at endpoint. Proportions of lean muscle mass and fat mass were measured with MRI technique. Each symbol represents an individual mouse (*n*=14 NH_L, *n*=14 AH_L, *n*=21 AH) and bars show medians. For statistical analysis 1way ANOVA Kruskal-Wallis test with Dunn's multiple comparisons test was performed. *$p<0.05$.
