# Supplementary material for: Early-Life Human Microbiota Associated With Childhood Allergy Promotes the T Helper 17 Axis in Mice
Source: Front Immunol. 2017 Dec 1;8:1699. doi: 10.3389/fimmu.2017.01699 (PMC5716970; doi:10.3389/fimmu.2017.01699)
Supplement: Supplementary file 3 [file Image_2.pdf]

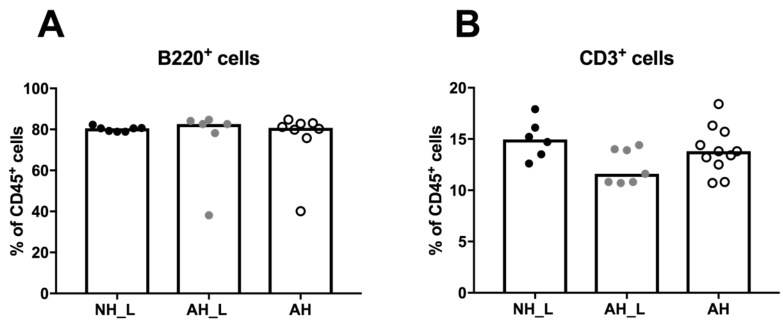

Supplementary Figure 2. Total lymphocyte percentages in Peyer's patches. Peyer's patches (PP) lymph nodes were isolated from small intestinal tissue, processed and the single cells were further characterized. (**A**) Proportion of B-cells, characterized as $B220^+$, within the $CD45^+$ cell population. Each symbol is equivalent to pooled material from 2 individual mice (*n*=7 NH_L, *n*=6 AH_L, *n*=8 AH). (**B**) Proportion of T-cells, characterized as $CD3^+$, within the $CD45^+$ cell population. Each symbol is equivalent to pooled material from 2 individual mice (*n*=6 NH_L, *n*=7 AH_L, *n*=11 AH). Bars represent median values.
