# Supplementary material for: Early-Life Human Microbiota Associated With Childhood Allergy Promotes the T Helper 17 Axis in Mice
Source: Front Immunol. 2017 Dec 1;8:1699. doi: 10.3389/fimmu.2017.01699 (PMC5716970; doi:10.3389/fimmu.2017.01699)
Supplement: Supplementary file 4 [file Image_3.pdf]

SUPPLEMENTARY FIGURES

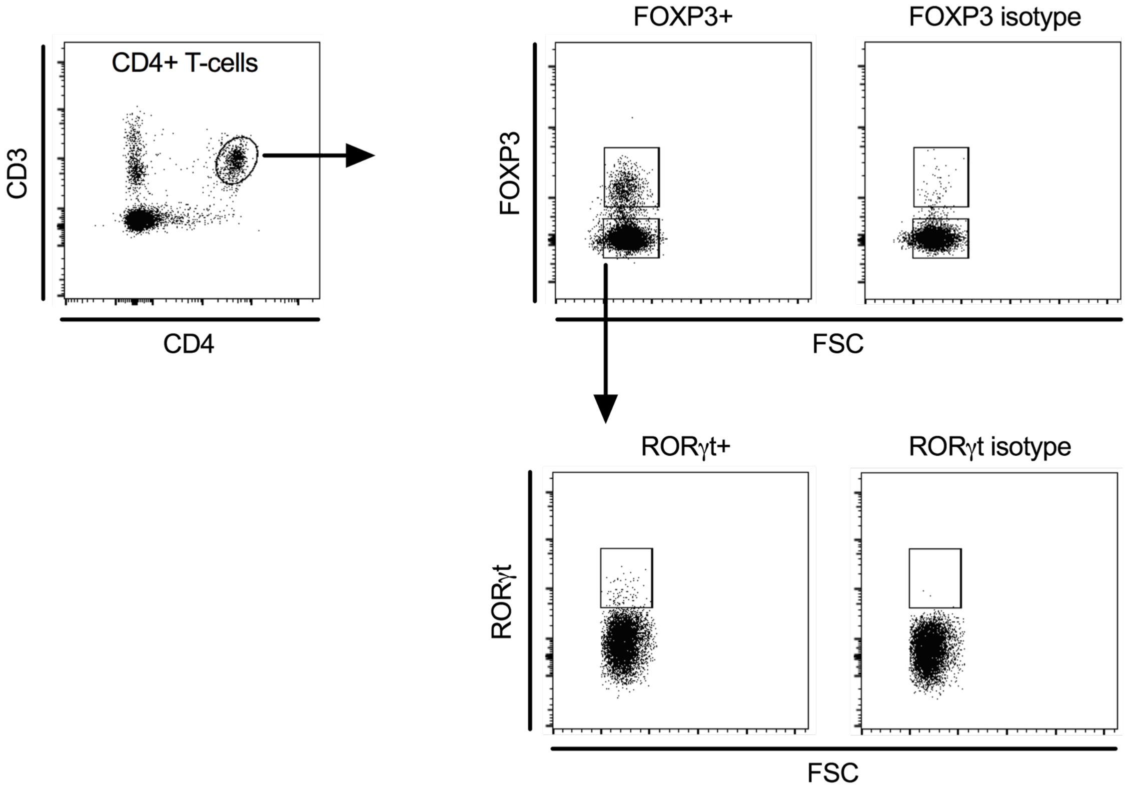

Supplementary Figure 3. FACS gating strategy for $CD4^+$ T-cells in PP. $CD4^+CD3^+$ T-cells were gated within $CD45^+$ lymphocytes. $FOXP3^-$ cells within the $CD4^+CD3^+$ population were further analyzed for RORγt expression.
