# Supplementary material for: Early-Life Human Microbiota Associated With Childhood Allergy Promotes the T Helper 17 Axis in Mice
Source: Front Immunol. 2017 Dec 1;8:1699. doi: 10.3389/fimmu.2017.01699 (PMC5716970; doi:10.3389/fimmu.2017.01699)
Supplement: Supplementary file 5 [file Image_4.pdf]

SUPPLEMENTARY FIGURES

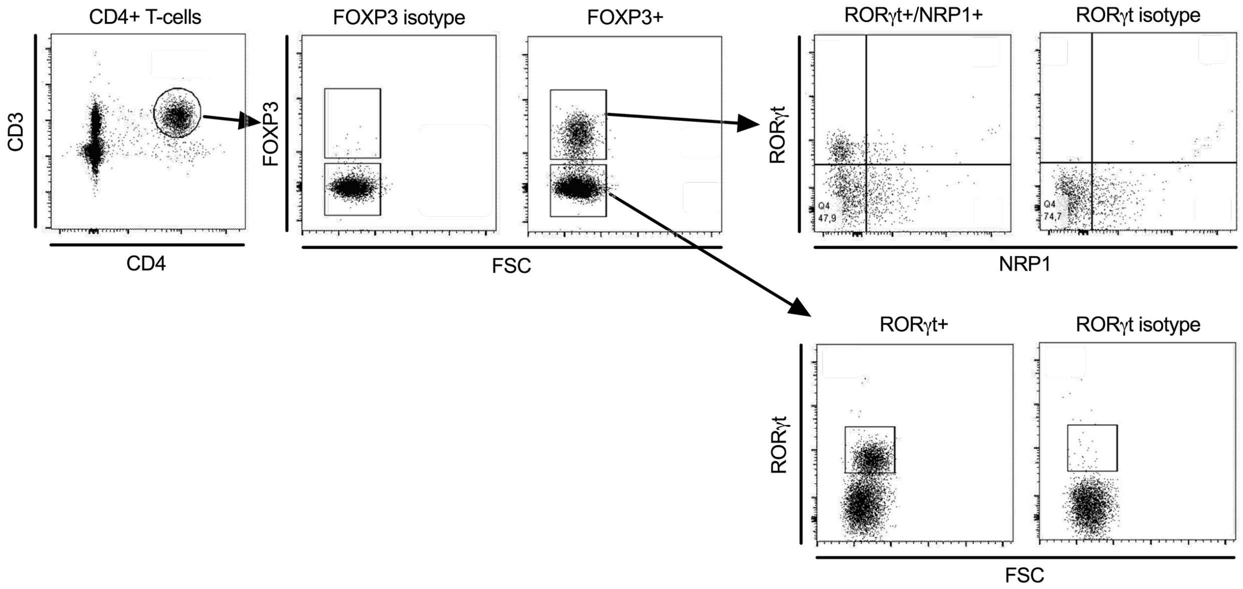

Supplementary Figure 4. FACS gating strategy for colonic lamina propria CD4$^+$ T-cells. CD4$^+$CD3$^+$ T-cells were gated within CD45$^+$ lymphocytes. FOXP3$^-$ cells within the CD4$^+$CD3$^+$ population were further analyzed for RORγt expression, while FOXP3$^+$ cells within the CD4$^+$CD3$^+$ population were further analyzed for RORγt and NRP1 expression.
