# Supplementary material for: Early-Life Human Microbiota Associated With Childhood Allergy Promotes the T Helper 17 Axis in Mice
Source: Front Immunol. 2017 Dec 1;8:1699. doi: 10.3389/fimmu.2017.01699 (PMC5716970; doi:10.3389/fimmu.2017.01699)
Supplement: Supplementary file 6 [file Image_5.pdf]

SUPPLEMENTARY FIGURES

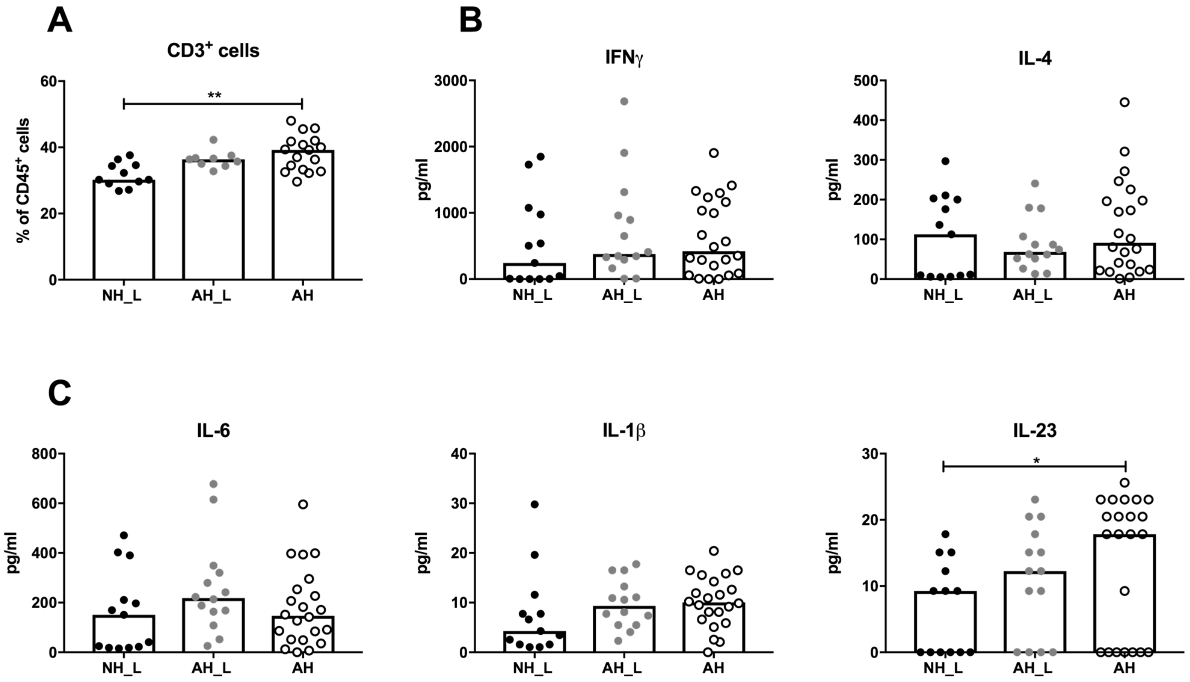

Supplementary Figure 5. Cytokine production in spleen. (**A**) The proportion of $CD3^+$ T-cells within the $CD45^+$ population (*n*=11 NH_L, *n*=9 AH_L, *n*=17 AH). (**B**) Systemic IFNγ and IL-4 in culture supernatants following stimulation of single cells from spleen with αCD3/αCD28 for 24h. (**C**) Systemic IL-6, IL-1β and IL-23 in culture supernatants following stimulation of single cells from spleen with αCD3/αCD28 for 24h. (for **B** and **C,** *n*=13 NH_L, *n*=14 AH_L, *n*=22 AH). Each symbol is equivalent to one individual mouse and bars represent median values. For statistical analysis, a 1way ANOVA Kruskal-Wallis test with Dunn's multiple comparisons test was performed. *p<0.05, **p<0.01.
