# Supplementary material for: Early-Life Human Microbiota Associated With Childhood Allergy Promotes the T Helper 17 Axis in Mice
Source: Front Immunol. 2017 Dec 1;8:1699. doi: 10.3389/fimmu.2017.01699 (PMC5716970; doi:10.3389/fimmu.2017.01699)
Supplement: Supplementary file 7 [file Image_6.pdf]

SUPPLEMENTARY FIGURES

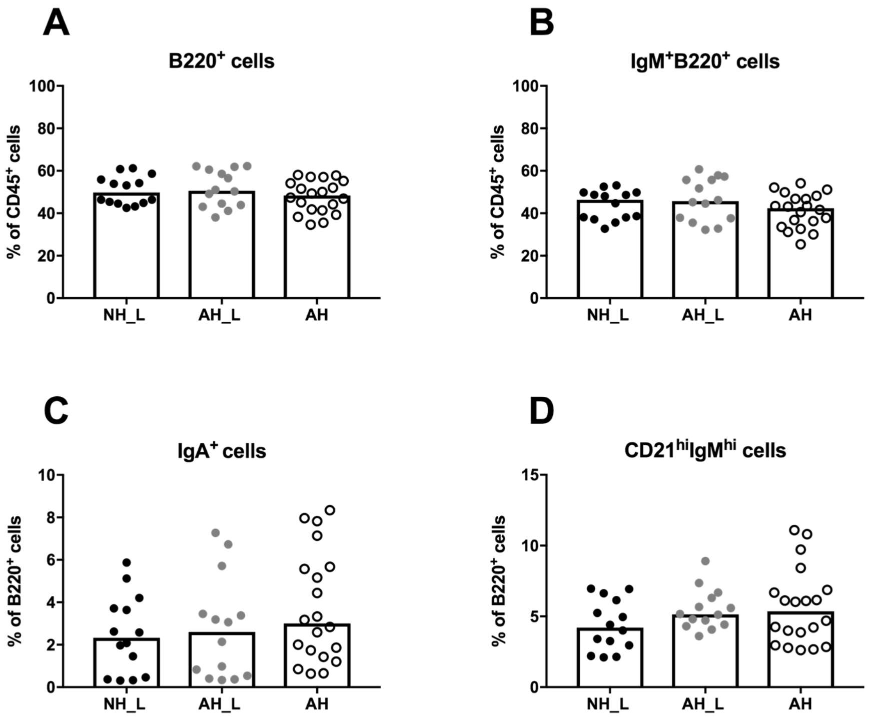

Supplementary Figure 6. B-cell populations in spleen. (**A**) Proportions of B-cells, characterized as B220$^+$ cells within the CD45$^+$ population (*n*=14 NH_L, *n*=14 AH_L, *n*=20 AH). (**B**) Proportions of IgM$^+$B220$^+$ cells within the CD45$^+$ population. **(C)** Proportion of IgA$^+$ B-cells within the B220$^+$ population. (**D**) The proportions of CD21^{hi}IgM^{hi} cells (marginal zone B-cells) within the B220$^+$ population (**B**-**D**: *n*=14 NH_L, *n*=14 AH_L, *n*=20 AH). Each symbol is equivalent to an individual animal and bars represent median values.
