# Supplementary material for: Early-Life Human Microbiota Associated With Childhood Allergy Promotes the T Helper 17 Axis in Mice
Source: Front Immunol. 2017 Dec 1;8:1699. doi: 10.3389/fimmu.2017.01699 (PMC5716970; doi:10.3389/fimmu.2017.01699)
Supplement: Supplementary file 8 [file Image_7.pdf]

SUPPLEMENTARY FIGURES

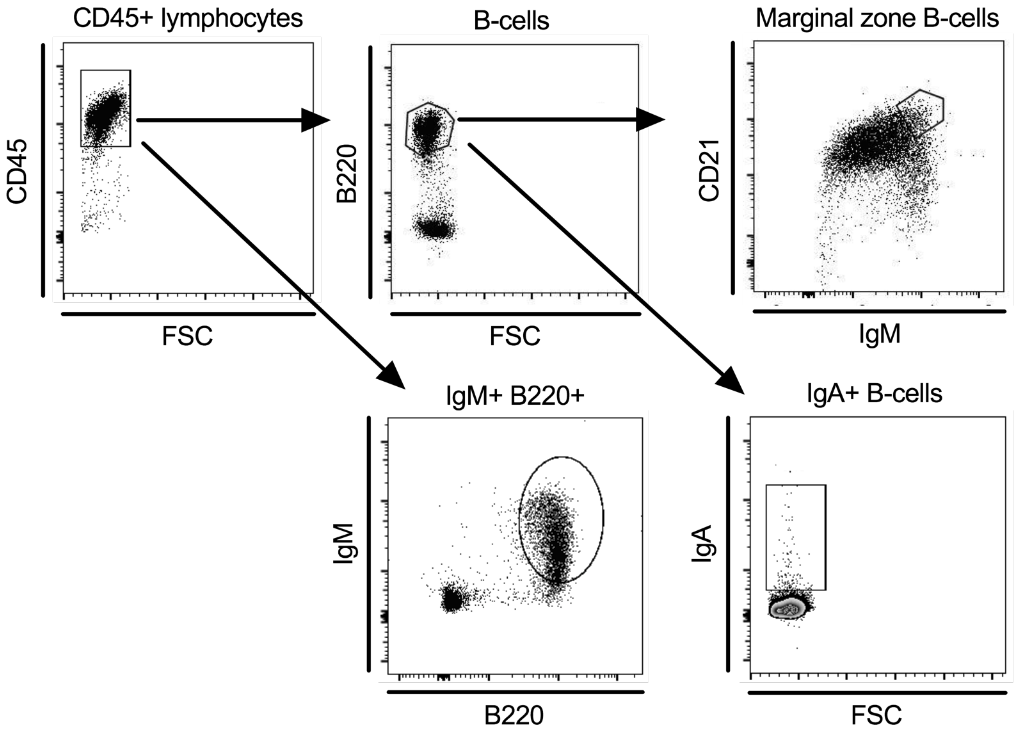

Supplementary Figure 7. FACS gating strategy for B-cells. B-cells were identified as B220^{+} cells within the CD45^{+} population. Marginal zone B-cells were defined as CD21^{hi}IgM^{hi}. IgM^{+}B220^{+} cells were gated within the CD45^{+} population. IgA^{+} B-cells were gated within the B220^{+} population.
