# Supplementary material for: Early-Life Human Microbiota Associated With Childhood Allergy Promotes the T Helper 17 Axis in Mice
Source: Front Immunol. 2017 Dec 1;8:1699. doi: 10.3389/fimmu.2017.01699 (PMC5716970; doi:10.3389/fimmu.2017.01699)
Supplement: Supplementary file 9 [file Image_8.pdf]

SUPPLEMENTARY FIGURES

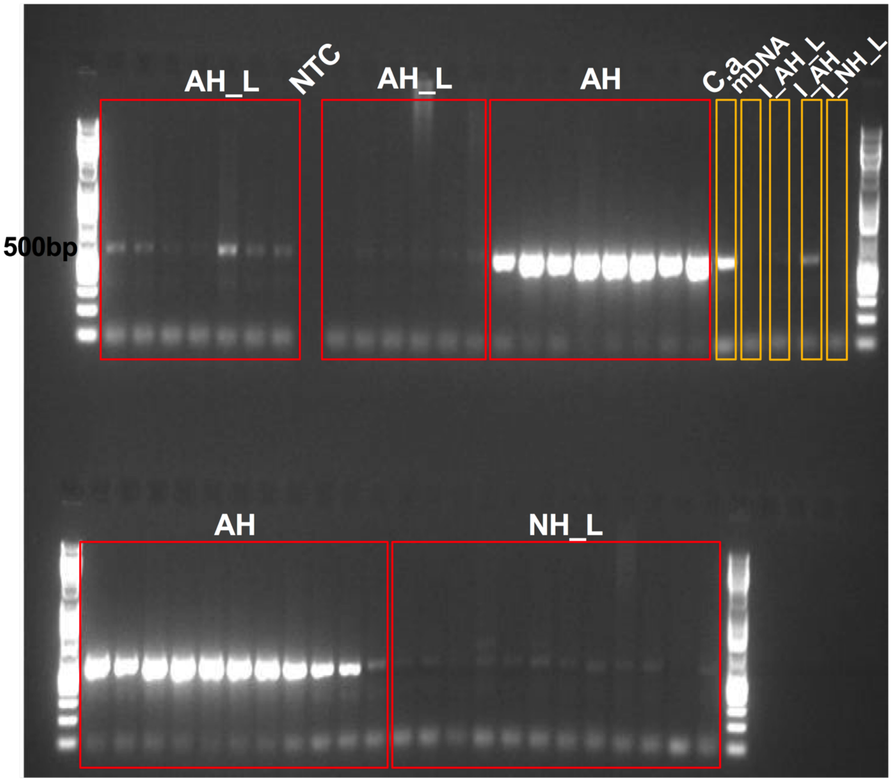

Supplementary Figure 8. PCR screening for fungal DNA. Agarose gel electrophoresis of PCR products amplified with general fungal primers ITS4 and ITS5. DNA extracted from cecal contents from animals in experimental groups AH_L, AH and NH_L was screened for fungal DNA. DNA samples extracted from the original infant fecal inoculum (I) for the different groups (I_NH_L, I_AH_L and I_AH) were included in the analysis. For controls no template control (NTC), genomic DNA from *C. albicans* (C.a.) and mouse (mDNA) were used. Each lane in the experimental groups represents an individual animal (*n*=12 NH_L, *n*=13 AH_L, *n*=19 AH).
